# Common contributing factors to COVID-19 and inflammatory bowel disease

**DOI:** 10.1016/j.toxrep.2021.08.007

**Published:** 2021-08-31

**Authors:** Ronald Neil Kostoff, Michael Brandon Briggs, Darja Kanduc, Darla Roye Shores, Leda Kovatsi, Alexander I. Vardavas, Alan L. Porter

**Affiliations:** a School of Public Policy, Georgia Institute of Technology, Gainesville, VA, 20155, United States; b Roscommon, MI, 48653, United States; c Dept. of Biosciences, Biotechnologies, and Biopharmaceutics, University of Bari, Via Orabona 4, Bari, 70125, Italy; d Department of Pediatrics, Division of Gastroenterology, The Johns Hopkins University School of Medicine, Baltimore, MD, 21287, United States; e Laboratory of Forensic Medicine and Toxicology, School of Medicine, Aristotle University of Thessaloniki, 54124, Greece; f Laboratory of Toxicology & Forensic Sciences, Faculty of Medicine, University of Crete, Greece; g R&D, Search Technology, Inc., Peachtree Corners, GA, 30092, United States; h School of Public Policy, Georgia Institute of Technology, Atlanta, GA, 30332, United States

**Keywords:** Pandemic, Coronavirus, COVID-19, SARS-CoV-2, LRDI, Severe acute respiratory syndrome, SARS-CoV, Inflammatory bowel disease, Ulcerative colitis, Crohn’s disease, Immune system dysfunction, Contributing factors

## Abstract

The devastating complications of coronavirus disease 2019 (COVID-19) result from an individual’s dysfunctional immune response following the initial severe acute respiratory syndrome coronavirus 2 (SARS-CoV-2) infection. Multiple toxic stressors and behaviors contribute to underlying immune system dysfunction. SARS-CoV-2 exploits the dysfunctional immune system to trigger a chain of events ultimately leading to COVID-19.

We have previously identified many contributing factors (CFs) (representing toxic exposure, lifestyle factors and psychosocial stressors) common to myriad chronic diseases. We hypothesized significant overlap between CFs associated with COVID-19 and inflammatory bowel disease (IBD), because of the strong role immune dysfunction plays in each disease.

A streamlined dot-product approach was used to identify potential CFs to COVID-19 and IBD. Of the fifty CFs to COVID-19 that were validated for demonstration purposes, approximately half had direct impact on COVID-19 (the CF and COVID-19 were mentioned in the same record; i.e., CF---→COVID-19), and the other half had indirect impact. The nascent character of the COVID-19 core literature (∼ one year old) did not allow sufficient time for the direct impacts of many CFs on COVID-19 to be identified. Therefore, an immune system dysfunction (ID) literature directly related to the COVID-19 core literature was used to augment the COVID-19 core literature and provide the remaining CFs that impacted COVID-19 indirectly (i.e., CF---→immune system dysfunction---→COVID-19).

Approximately 13000 potential CFs for myriad diseases (obtained from government and university toxic substance lists) served as the starting point for the dot-product identification process. These phrases were intersected (dot-product) with phrases extracted from a PubMed-derived IBD core literature, a nascent COVID-19 core literature, and the COVID-19-related immune system dysfunction (ID) core literature to identify common ID/COVID-19 and IBD CFs. Approximately 3000 potential CFs common to both ID and IBD, almost 2300 potential CFs common to ID and COVID-19, and over 1900 potential CFs common to IBD and COVID-19 were identified. As proof of concept, we validated fifty of these ∼3000 overlapping ID/IBD candidate CFs with biologic plausibility. We further validated 24 of the fifty as common CFs in the IBD and nascent COVID-19 core literatures. This significant finding demonstrated that the CFs indirectly related to COVID-19 -- identified with use of the immune system dysfunction literature -- are strong candidates to emerge eventually as CFs directly related to COVID-19. As discussed in the main text, many more CFs common to all these core literatures could be identified and validated.

ID and IBD share many common risk/contributing factors, including behaviors and toxic exposures that impair immune function. A key component to immune system health is removal of those factors that contribute to immune system dysfunction in the first place. This requires a paradigm shift from traditional Western medicine, which often focuses on treatment, rather than prevention.

## Highlights

• Validated fifty contributing factors (CFs) in common between COVID-19 and Inflammatory Bowel Disease (IBD).
• Method allows identification of directly related CFs to COVID-19 from indirectly related CFs to COVID-19.
• Many more CFs common to COVID-19 and IBD are possible with present approach.
• Results provide the basis for unified theory of chronic-infectious diseases.

## Graphical abstract

**Figure:**
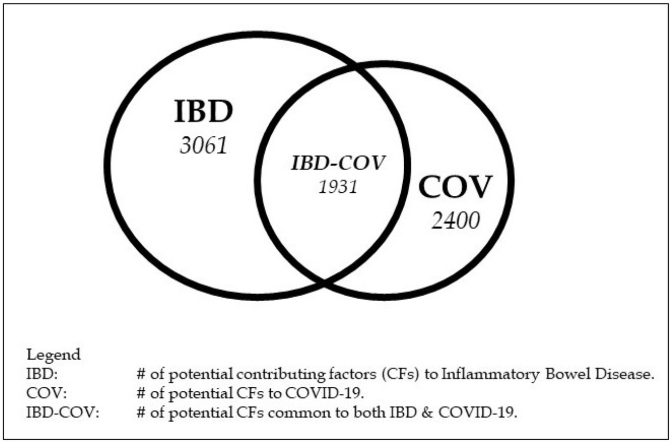

## 1 Introduction

The present study aims to demonstrate commonality between the contributing factors (CFs) to COVID-19 and Inflammatory Bowel Disease (IBD), and show that the bases for these superficially different diseases have important similarities. Much of the underlying motivation for this study has been presented previously [1], and will not be repeated here.

The virus associated most closely with COVID-19 (SARS-CoV-2) is transmissible. Whether serious consequences occur from this transmission depends on the health of the host’s immune system [[2], [3], [4]]. In our model, these serious consequences of COVID-19 result from the effective exploitation of a dysfunctional immune system by the SARS-CoV-2 virus. In this exploitive process, genetic disposition and real-life exposures to multiple toxic stressors, and toxic behaviors, lay the ground work for immune system dysfunction [5]. Following SARS-CoV-2 exposure, the dysfunctional immune system is unable to neutralize the SARS-CoV-2 virus, thereby allowing the virus to enter and replicate in the cell and trigger a chain of events ultimately leading to COVID-19 [2,3].

If immune system dysfunction is a/the major factor in the severity of both infectious and chronic diseases, then a necessary, but not necessarily sufficient, condition for prevention and successful longstanding treatment is elimination of those factors that contribute to the dysfunction of the immune system. The virology-centric approach used currently for COVID-19 reflects damage control for a dysfunctional immune system (*e.g.*, quarantine, face masks, vaccines, anti-viral treatments, *etc.*). A toxicology-centric approach would be aimed at identifying and removing the CFs to immune system dysfunctionality, whose evidentiary basis would require going beyond current single-stressor laboratory experiments to more comprehensive stressor combination experiments [2,6]. We hypothesize that COVID-19 and IBD (a chronic inflammatory disease associated with immune dysfunction) share similar CFs.

## 2 What is novel in our study

There are three main components to the study. The first is use of the dot-product approach to streamline the identification of candidate CFs to both IBD and COVID-19. These CFs impact IBD directly (the candidate CF is located in the same record as IBD: e.g., “chronic alcoholism exacerbates ulcerative colitis”) and impact COVID-19 directly and indirectly (the candidate CF is located in a non-COVID-19 core literature record linked closely to COVID-19: e.g., “chronic alcoholism exacerbates immune system dysfunction”, where ‘immune system dysfunction’ has been shown in the COVID-19 core literature to be closely related to COVID-19). Candidate CFs identified by the dot-product approach are then validated as actual CFs. The dot-product approach is described in more detail in the Methodology section.

Second is the use of a literature related to COVID-19 for the purpose of identifying candidate CFs to COVID-19 that were related indirectly. At the time the data retrieval and analysis were performed, the COVID-19 core literature was quite new and growing rapidly. As shown later in this paper, it was essentially a literature focused on reaction to, and containment of, the pandemic. Insufficient time had elapsed for a large number of studies to be performed and reported that could link toxic exposures and behaviors to COVID-19 directly, although there were some such studies performed and reported. Therefore, a mature literature related directly to COVID-19 was required to allow toxic exposures and behaviors to be linked to COVID-19 indirectly through their direct link to the more mature literature. Since one of the main characteristics of COVID-19 (as evidenced in its core literature) is dysfunction of the immune system, we selected the immune system dysfunction literature as the mature literature directly related to COVID-19. Toxic stimuli and behaviors that impacted the immune system adversely to cause impairment and dysfunction were evaluated as candidate CFs that impacted COVID-19 indirectly. As will be shown in the results section, approximately half of the CFs related indirectly to COVID-19 were also related directly to COVID-19. This strong overlap confirms the validity of using an appropriate literature directly related to COVID-19 to augment identification of potential CFs to COVID-19. Since we identified the CFs related indirectly to COVID-19 prior to identifying the CFs related directly to COVID-19, this means our approach was effective for identifying potential direct impact CFs. The ability to identify potential direct impact CFs from the identification of indirect impact CFs can be exploited to initiate new research projects to confirm the transmutation of indirect impact CFs to direct impact CFs, and should be of high interest to researchers, research managers, research sponsors, and venture capitalists.

Third is the demonstration that the direct and indirect CFs to COVID-19 overlapped with the direct CFs to IBD.

**Table:** 

This extensive overlap means that 1) many (if not most) of the causes for both diseases are similar, 2) any methods for prevention would have to be similar, and 3) at least some (if not many/all) methods for reversal would have to overlap. That is a result/conclusion of the highest significance and has profound significance for how both types of disease are treated/prevented.

Given that the present approach to controlling the pandemic is essentially virology-based, with essentially no component addressing the toxicology, there is a major mismatch between what is needed to control the pandemic and what is being done.

## 3 Commonality of CFs TO IBD and COVID-19

### 3.1 Background

Our group has been developing protocols to prevent and reverse chronic diseases [7,8]. The central component of our approach is identification and elimination of CFs to these myriad chronic diseases. The question arises: can our toxicology-based approach for preventing and reversing chronic diseases be integrated successfully with the present virology-based approach to preventing and reversing communicable diseases that exploit immune system dysfunction, such as COVID-19? Before we demonstrate our integrated virology-toxicology approach for IBD-COVID-19, we present a brief summary of the key components: COVID-19, IBD, Toxicology.

### 3.2 COVID-19

Over the past two decades, there have been at least three major coronavirus-based infectious disease outbreaks/epidemics/pandemics: Severe Acute Respiratory Syndrome (SARS), 2002–2003; Middle East Respiratory Syndrome (MERS), starting in 2012; and COVID-19, starting in December 2019. There are a number of similarities among these three infectious diseases, including abnormal values of selected inflammatory biomarkers (*e.g.*, neutrophils, lymphocytes, albumin, CRP, TNF-alpha, *etc.*), and pulmonary inflammation/damage. Another important similarity among these infectious diseases is the demographic affected most severely: the elderly and others who have comorbidities associated with dysfunctional immune systems [1,[9], [10], [11], [12], [13]].

### 3.3 IBD

IBD is a spectrum of chronic inflammatory gastrointestinal (GI) disorders, most commonly categorized as Crohn’s disease (CD) and ulcerative colitis (UC) [14]. The incidence of IBD is rising in industrialized countries and in children, suggesting exposures to CFs are at play [15,16]. The pathogenesis of IBD is multifactorial. While multiple genes associated with dysregulation of both innate and adaptive immunity have been identified, environmental factors, including diet, infection, and toxin exposure that alter the intestinal microbiome, also trigger epigenetic alterations in immune regulation [17,18]. Treatment often includes immunosuppressive therapy, which may increase the risk of serious infection. Of particular interest, a large international registry from 49 countries of patients with IBD has found that those patients treated with biologics, such as anti-tumor necrosis factor, had lower rates of severe COVID-19 [19]. It was postulated that biologic therapies suppress the cytokine storm associated with severe COVID-19. The same protection was not seen with other immunosuppressive medications, including thiopurines and corticosteroids [19].

### 3.4 Toxicology

In its broadest sense, toxicology is the study of the impact that toxic stimuli and toxic behaviors, and their combinations, can have on all members of the animal kingdom and their environment. Its two most important components are epidemiological-type studies to identify potential adverse effects of candidate toxic stimuli and behaviors, and laboratory studies to identify mechanisms that link the stimuli to their adverse effects. The toxic stimuli/behavior exposures can range from acute to chronic, and the doses can span a wide spectrum.

Toxicological components constitute the bulk of modifiable CFs responsible for IBD and COVID-19. The toxicological components included in the present study cover toxic lifestyles (diet, activity, sleep, substance abuse, etc.), medical procedures (drugs, diagnostics, surgery, non-drug therapies, etc.), bio-organisms (fungi, mold, parasites, viruses, bacteria, etc.), environments, occupations, psychosocial events, and socioeconomic environments. The laboratory-based evidence for the toxicity of most toxic substances is obtained through single-stressor laboratory experiments, which under-represent real-world effects. Combinations of toxic stimuli reflect real-world exposures, and doses of substances that can cause damage in combinations are lower than doses that can cause damage in single-stressor experiments of those substances [6]. Each of these factors plays a key role in such chronic exposure paradigms, revealing the importance of required further toxic evaluations so as to discover possible routes that would eventually lead to a human risk and even to a specific link between an epidemic and a disease.

The rapidly growing body of scientific evidence on COVID-19 indicates that in order for a patient to exhibit serious symptoms and side-effects, an underlying dysfunction of the immune system is necessary. Different factors, among which is the genetic disposition, as well as the exposure to toxic stimuli, aid the virus in rendering the immune system vulnerable [5].

Our results address a wide range of toxic stimuli; a couple that have wide impact are mentioned here briefly. One of the toxic stimuli that plays an important role seems to be excessive alcohol consumption. Recent work implies that it affects the immune system in a way that renders it more vulnerable to SARS-CoV-2 virus infection and that the underlying mechanism includes the reduction of T lymphocytes, the increase of proinflammatory cytokines, the diminished function and number of natural killer cells and the inadequate function of the macrophages [20]. It has also been proposed that chronic ethanol consumption leads to malnutrition and lack of micronutrients that are necessary for the normal function of the immune system [21].

Another toxic stimulus that is attracting attention (as to its implication in SARS-CoV-2 virus infection susceptibility) is smoking. Because of conflicting evidence of smoking impact on both IBD and COVID-19, it was not included as a CF in our results, but is addressed in detail in the Discussion section.

### 3.5 Identification of CFs common to IBD and COVID-19

#### 3.5.1 Overview

A credible identification of CFs common to two diseases (such as IBD and COVID-19) based on their core literatures depends on three major criteria: the relative size (number of records) of the literatures; the relative age of the literatures; the relative thrusts of the literatures. The closer these core literatures are matched based on the three criteria, the more uniform the basis for identifying CFs common to the two diseases.

#### 3.5.2 IBD/COVID-19 core literatures

The IBD core literature used for the present study spanned the timeframe 1990–2020, consisted of ∼75,000 records, and covered a wide variety of topics including treatments, causes, and mechanisms. The COVID-19 core literature we retrieved initially (mid-December 2020) was focused strictly on COVID-19 and SARS-CoV-2. We did not include coronaviruses in general, MERS, SARS, or other related issues. While the initial documents of this focused literature were published in December 2019, the rates of articles published did not dramatically accelerate until the May-June 2020 time frame. Therefore, the bulk of the COVID-19 database we retrieved (as of mid-December 2020) was six months old, or less.

#### 3.5.3 COVID-19 core literature deficiencies for identifying directly related CFs

We read thousands of COVID-19 core literature record titles to identify topics of interest. The main emphases of the COVID-19 core literature titles are: 1) containing the pandemic; 2) identifying the major abnormal biomarker values and symptoms of people hospitalized with COVID-19; 3) repurposing and testing treatments; 4) developing and testing vaccines; 5) assessing the effects of the pandemic on behaviors, medical treatments and procedures; and 6) reviews of treatments, vaccines, restrictions, etc. In short, the COVID-19 core literature is mainly focused on containment rather than prevention!

There has been insufficient time to conduct the lengthy laboratory experiments required to link CFs to COVID-19 or conduct the longer-term epidemiological studies required to show these relationships. As a result, the number of CFs related directly to COVID-19 in this nascent literature would be expected to grossly underestimate the number of CFs that actually impacted COVID-19 directly in the real world. Therefore, a more mature literature directly related to the COVID-19 core literature, which includes the longer-term studies that can demonstrate links of CFs immune system dysfunction consequences, is required to augment the core COVID-19 literature for the purpose of identifying CFs directly and indirectly related to COVID-19.

#### 3.5.4 Augmented COVID-19 core literature for identifying CFs indirectly related to COVID-19

There are many characteristics (specific and general biomarkers, symptoms, related diseases, etc.) of the COVID-19 core literature that could serve as a basis for identifying literatures directly related to the COVID-19 core literature. We reviewed many articles in the nascent COVID-19 core literature, and concluded that the dominant characteristic of the COVID-19 core literature was widespread dysfunction of the immune system. Therefore, we selected the non-COVID-19 immune system dysfunction literature (ID) as the directly related literature to the COVID-19 core literature that would augment the identification of CFs directly impacting COVID-19 using the COVID-19 core literature. CFs to immune system dysfunction obtained from analysis of the ID literature were linked indirectly to COVID-19 through the multi-linked path CF----→immune system dysfunction----→COVID-19.

The size of the ID literature depended upon the time frame selected. For any timeframe of interest, it was about three times the size of the IBD core literature. Thus, a tradeoff between timeframe and size was necessary for the ID literature. It was decided the maturity of the literature was higher priority than size, so the ID literature was selected to cover the same timeframe as the IBD literature. The ID literature selected spanned 1990-2020 (the same timeframe as the IBD core literature), and consisted of ∼200,000 records. Because of the discrepancy in size between the IBD core literature (∼75,000 records) and the ID core literature, we expected that IBD/ID overlaps would be limited by the potential CFs in the IBD literature, which in fact was the result. We determined commonality between CFs to IBD and COVID-19 using a streamlined dot-product approach (intersection of phrase lists) and we identified modifiable factors that contribute to both IBD and COVID-19.

#### 3.5.5 Thematic differences between ID and COVID-19 core literatures

The metrics used to evaluate thematic differences between the core ID and COVID-19 literatures were the main specific biomarkers and the abnormalities of their values. We examined the main specific biomarkers exhibiting abnormalities in the COVID-19 core literature, and found strong overlap with the main specific biomarkers in the ID literature. The major difference was the priority ranking of the biomarkers, as measured by their record frequency occurrences. The main COVID-19 core literature biomarkers and directions of value change reflected the response of an already dysfunctional immune system, with stronger emphasis on the inflammation immune biomarkers relative to the oxidative stress biomarkers. However, the presence of biomarkers in the COVID-19 core literature reflecting low oxygen (hypoxia) and higher coagulation suggested that oxidative stress played an important role in the course of the disease [22]. More broadly, “Oxidative stress by reactive oxygen species (ROS) is related to all the main changes observed in other inflammatory and infectious diseases and could be the connecting point that unites all these events [22]”.

The main ID core literature biomarkers and directions of value change reflected evolving immune system function, with more balance between the inflammation biomarkers and the oxidative stress biomarkers. In the ID core literature, the toxic substances and behaviors that drove the immune system dysfunctional were indirect CFs to COVID-19 through a two-step linkage. In the COVID-19 core literature, the toxic substances and behaviors related to COVID-19 were direct CFs to COVID-19. The IBD core literature biomarkers were more weighted towards the inflammation immune biomarkers relative to the oxidative stress biomarkers.

What are the specific types of immune system dysfunction that enabled the COVID-19 response? These can be gleaned from the myriad comorbidities and chronic diseases associated especially with the more severe consequences from COVID-19. As our past studies on specific chronic diseases have shown, there are many CFs for each disease that impact the immune, neural, endocrine, and circulatory systems. The ID core literature selected for this study includes myriad CF-comorbidity-immune dysfunction linkages. Because of these linkages, the CFs in the ID literature contribute in large part to the immune dysfunction that enables the emergence of serious COVID-19 consequences. As the COVID-19 literature expands and matures, and begins to incorporate those CFs that produce the COVID-19-enabling dysfunctionality, we expect the gap between the ID-IBD common CFs and the COVID-19-IBD common CFs to narrow substantially.

#### 3.5.6 Update of COVID-19 core literature

In the initial phases of the study, we decided to select fifty candidate CFs and validate them and their commonality in the IBD and ID core literatures. These candidate CFs would be related indirectly to COVID-19 because only the ID literature was used. Because of the nascency of the COVID-19 core literature, and the relatively few directly related CFs, we did not include these directly related CFs in the IBD/ID CF comparison. However, we performed periodic updates of the COVID-19 core literature because of its extremely rapid growth. As a result, we noticed an increase in the number of CFs from the group of fifty that were related directly to COVID-19. By late January 2021, we decided to include the CFs directly related to COVID-19 in the comparison of common IBD/ID CFs, and we further updated the CFs directly related to COVID-19 at the end of the study. The important point here is that all fifty CFs directly related to IBD and indirectly related to COVID-19 were selected and validated prior to the decision to ascertain how many of the fifty were directly related to COVID-19.

Updating the COVID-19 literature in late March 2021 allowed significant identification of CFs directly related to COVID-19 that had been identified previously as indirectly related to COVID-19.

**Table:** 

Thus, the presence of a significant number of CFs that became directly related to COVID-19 in the biomedical literature in 2020 showed that the ID literature could be used to identify indirectly-related CFs to COVID-19 that had promise of becoming directly related CFs as the core COVID-19 literature expanded and incorporated more CF identification studies.

This proof-of-principle demonstrates that CFs identified initially as indirectly related to COVID-19, using an appropriate literature directly related to COVID-19, had good promise of becoming CFs directly related to COVID-19 after relevant studies had been performed. Thus, there exists the potential for the many hundreds of CFs indirectly related to COVID-19 identified in this study to be confirmed as CFs directly related to COVID-19 as the COVID-19 core literature grows and matures to incorporate future CF identification studies.

### 3.6 Myriad commonalities between IBD and ID

In 2014, the first author published a study showing theme commonalities between Parkinson’s Disease (PD) (neurodegenerative) and Crohn’s Disease (autoimmune) using phrase matching and bibliographic coupling (shared references) between the two disease literatures [23]. Because of the strong emphasis on shared references, the commonality of PD and CD at a more fundamental mechanism level was demonstrated. Combining these two approaches for identifying commonality (CF commonality and bibliographic coupling/phrase matching) could provide deeper understanding at different levels of commonality between IBD and ID/COVID-19.

## 4 Methodology

### 4.1 Dot-product approach

The streamlined method used for this study is termed a dot-product approach [1]. Lists of known toxic substances were aggregated from myriad (mainly) government agencies, and combined with lists of CFs identified in our previous disease studies. This produced a final database of approximately 13000 potential CFs to disease. While this is certainly a large number of potential CFs, it undoubtedly omits additional CFs that a well-resourced study could have identified.

A core literature query was defined for IBD, applied to PubMed, and the resultant retrieval (∼75000 records with abstracts, covering 1990-2020) was imported into VantagePoint (VP) software (www.theVantagePoint.com). The title and abstract phrases of the retrieved records were parsed, resulting in lists of many phrases. The same procedure was followed for the ID core literature (∼202000 records with abstracts, covering 1990-2020), and for the COVID-19 core literature (∼54000 records with abstracts, covering 1 December 2019-mid-December 2020, and periodically updated to include about 84,000 records by the end of March 2021).

The external list of ∼13000 phrases of potential CFs was intersected with the parsed list of abstract phrases in the IBD, ID, and COVID-19 core literatures to generate the sub-set of the ∼13000 phrases relevant to each core literature. There were ∼3100 candidate IBD CFs, ∼6500 candidate ID CFs, and ∼2400 candidate COVID-19 CFs (candidate means they are potential CFs, but need to be validated as actual CFs). These intersected lists were compared, and the candidate CFs in common between IBD and ID were identified initially. When the decision was made to include CFs that directly impacted COVID-19, then the candidate CFs in common between IBD and COVID-19, and ID and COVID-19, were identified. Approximately 3000 candidate CFs in common between IBD and ID, 1900 candidate CFs in common between IBD and COVID-19, and 2300 candidate CFs in common between ID and COVID-19 were identified, albeit some being variants of the same concept.

**Fig. 1:**
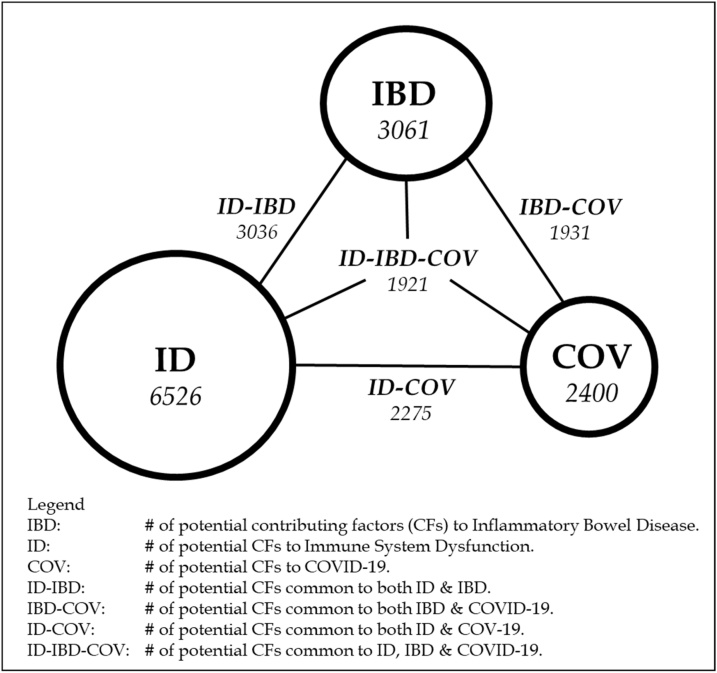
Potential common contributing factors among IBD, ID, and COVID-19.

These CF commonalities are displayed graphically in Fig. 1. The node values (within the circles) are the CFs obtained from each core literature using the dot-product approach, and the link values are the common CFs for the diseases at the link’s terminus points. The entry at the center of the triangle (ID-IBD-COV) reflects the CFs common to all three diseases.

The commonality of the two mature core literatures (ID-IBD) is limited strongly by the number of CFs in the smaller IBD literature, and the near-identical number of IBD CFs and common IBD-ID CFs reflects the IBD literature’s capture of some fraction of the inflammation component of the ID core literature. A similar relationship holds for ID-COV commonality, with perhaps more oxidative stress component capture of the ID core literature compared to the IBD capture fraction. The largest distinction is between the IBD and COV literatures, reflecting the moderately larger role of oxidative stress in the COV literature signified by the hypoxia and coagulation biomarkers mentioned previously [22]. Finally, the CFs common among all three diseases (ID-IBD-COV) are limited by, and essentially identical to, the IBD-COV commonality. This is to be expected, given that ID-IBD and ID-COV commonalities were limited by (and almost identical to) the number of IBD and COV CFs, not by numbers of ID CFs. However, this is a very conservative estimate of candidate CFs in common for both comparisons, for the reasons presented in the next section.

### 4.2 Limitations of dot-product approach

First, only CFs that occurred within the IBD and ID core literatures (direct impact CFs), or within the IBD and COVID-19 core literatures, were used for the dot-product. Thus, if a candidate CF was shown to enhance oxidative stress, and enhanced oxidative stress was a marker of IBD or ID, or of IBD or COVID-19, the candidate CF became a confirmed direct impact CF if the article that showed the linkage to CF was in the IBD or ID core literature, or the IBD or COVID-19 literature, respectively. If the candidate CF enhanced, say, “oxidative stress,” but the article(s) that showed this linkage was not in the IBD or ID core literature, or the IBD or COVID-19 core literature, then the candidate CF did not become a validated direct impact CF. Given that “oxidative stress” occurs in about 800 abstracts in the IBD literature used for the present study, and about 230000 abstracts in the total Medline database, these indirect impacts that require merging of two records (impact of CF on oxidative stress; impact of oxidative stress on IBD) have the potential to expand the number of records in common substantially. If indirect impacts, as defined above, had been included, much larger numbers of both CFs and commonalities would have resulted [1].

Second, all the matching and dot-product operations required *exact phrase matching*. The slightest difference between any two phrases meant neither phrase survived the dot-product process. Given the disagreements on phrase representations among the toxic substance list providers for identical concepts, especially chemical formulas, and the subsequent disagreements between the external phrases and the parsed phrases of the PubMed retrievals, we estimate many hundreds (or more) of phrases in common were lost. The fact that ∼3000 common IBD and ID phrases, and ∼1900 common IBD and COVID-19 phrases, survived is testament to the potentially large commonality between the CFs to IBD and COVID-19. We would expect similar results between COVID-19 and most (if not all) chronic diseases, based on the findings in our Pervasive Causes of Disease eBook [1,24].

Third, as has been shown in past studies [6,[25], [26], [27]], there are myriad substances and radiation forms that, at specified dosages, exhibit toxic effects only when combined with other toxic substances and radiation forms. Most of these low-dose CFs would not be identified in laboratory experiments, since the laboratory experiments tend to focus on single stressor results. If such combination laboratory experiments were to be performed, we would expect the number of CFs to increase substantially, given the total number of combinations possible [6].

Fourth, in order for a toxic substance or behavior to have been included in the core literature for IBD or ID or COVID-19, it had to have been researched and reported in Medline. Given the large number of potentially toxic substances and behaviors possible [24], and the limited number of biomarkers used in experiments to identify their adverse effects, the number of toxic combinations studied is severely limited [25]. Therefore, the number of potential CFs that enter any disease core literature is also limited [1].

Additionally, there are many types of documentation other than journals indexed in Medline (or the Science Citation Index), such as books, Web documents, less-well-known indexing services, more obscure journals, *etc.* This also would limit CFs accessible to the present study, which focused mainly on Medline records.

### 4.3 Selection of candidate common CFs for validation

The phrases in common between IBD and ID, and between IBD and COVID-19, should be viewed as candidate CFs, which must be validated as actual CFs by detailed analysis. How many validated CFs are required to support the hypothesis of common causation between the two diseases? There are two main criteria to be considered in making the selection. The first criterion is numbers of CFs in common. The second criterion is the importance of the CFs in contributing to the disease.

If, as in many large and complex systems, the system operation is determined mainly by a few significant factors, then a handful of such significant factors is all that would be required to support our hypothesis. If no such significant factors stand out, then more CFs would be required to support the hypothesis of common cause.

For IBD and ID, there were significant factors that stood out, and these were the foundation of the validation selection process. A balance/tradeoff between the two major selection criteria resulted in the selection of fifty common phrases between IBD and ID to be validated as CFs. These fifty included those deemed most significant and spanning the five-category taxonomy we have developed for classifying modifiable CFs to disease: Lifestyle, Iatrogenic, Biotoxins, Occupational/Environmental, PsychoSocial/ SocioEconomic [7]. We did not include Genetics, since the CFs in our definition are viewed as modifiable, meaning they are somewhat under our control.

After the fifty CFs common to IBD and ID had been selected and validated, we decided to ascertain which of them were valid COVID-19 direct impact CFs and thereby also common with IBD

## 5 Results and discussion

### 5.1 Results

The fifty CFs in common between IBD and ID selected for validation, as well as the 24 direct impact CFs in common between IBD and COVID-19, are presented in Table 1. The detailed record excerpts showing these linkages are presented in Appendix A.

**Table 1:** Common contributing factors to IBD and immune degradation/COVID-19.

Contributing Factors	Category	References		
		IBD	ID	COV
Advanced glycation end products	1	[79]	[80]	[81]
Alcoholism	1	[82]	[83]	[84]
Carrageenan	1	[85]	[86]	
Consumer antimicrobials	1	[87]	[87]	
High-fat diet	1	[88]	[89]	[90]
High-salt diet	1	[91]	[92]	
Malnutrition	1	[93]	[94]	[95]
Maternal smoking	1	[96]	[97]	[98]
Nitrosamines	1	[99]	[100]	
Polysorbate-80	1	[101]	[102]	
Refined carbohydrates	1	[103]	[104]	[104]
Sedentary	1	[105]	[106]	[107]
Vitamin D deficiency	1	[108]	[109]	[110]
Antibiotics	2	[111]	[112]	
Cisplatin	2	[113]	[114]	
Opioids/Morphine	2	[115]	[116]	[117]
Oral contraceptives	2	[118]	[119]	
Radiotherapy	2	[120]	[121]	[122]
Renal transplantation	2	[123]	[124]	[125]
Rituximab	2	[126]	[127]	[128]
Vaccines	2	[129]	[130]	
Cytomegalovirus	3	[131]	[132]	[133]
N-Ethyl-N-Nitrosourea	3	[134]	[135]	
Trichothecenes	3	[136]	[137]	
Zearalenone	3	[138]	[138]	
Air Pollutants	4	[139]	[140]	[141]
Aluminum	4	[142]	[143]	
Benzene	4	[144]	[145]	[146]
Benzo(A)Pyrene	4	[147]	[148]	
Bisphenol	4	[149]	[150]	[151]
Cadmium	4	[152]	[153]	[154]
Chlorpyrifos	4	[155]	[156]	
Fluoridated water	4	[157]	[158]	
Mercury	4	[159]	[160]	[154]
Microplastics	4	[161]	[161]	
Nickel	4	[162]	[163]	
Paraquat	4	[164]	[165]	
PCB (polychlorinated biphenyls)	4	[166]	[167]	
PFOA (perfluoroctanoic acid)	4	[168]	[169]	[170]
Polycyclic aromatic hydrocarbons	4	[171]	[172]	[173]
Silica dust	4	[174]	[175]	[176]
Sodium dichromate dihydrate	4	[177]	[178]	[179]
Titanium dioxide	4	[180]	[181]	
Triclocarban	4	[182]	[183]	
Turpentine	4	[184]	[185]	
Low socioeconomic status	5	[186]	[187]	[188]
Posttraumatic stress	5	[189]	[190]	
Psychological stress	5	[191]	[192]	[193]
Restraint stress	5	[194]	[195]	
Sexual abuse	5	[196]	[197]	

Table 1 contains five columns. The first (leftmost) column (CF) is the CF that was validated. The second column contains the category to which the CF is assigned (1 = lifestyle; 2 = iatrogenic; 3 = biotoxin; 4 = occupational/environmental; 5 = psychosocial/socioeconomic). The third, fourth, and fifth columns contain the references that link each CF to the biomarkers, and are presented in the order of IBD literature, ID literature, and COVID-19 literature (the latter only for the 24 selected for validation from the COVID-19 literature).

Table B1, contained in Appendix B, is similar in content to Table 1, with the exception of additional columns for biomarkers impacted in establishing the linkage between the CF under consideration and the disease (IBD, ID, COVID-19). There are five columns in Table B1. The first column contains the CFs. The second column contains the CF category. The third column contains the disease impacted. The fourth column contains the biomarkers impacted by the CFs for each disease, and the fifth column contains the reference showing the linkage between the CF and the disease. Examination of the biomarkers listed in Table B1 shows the strong emphasis on immune system biomarkers, mainly inflammation-centric with some emphasis on oxidative stress biomarkers.

### 5.2 Discussion

The results show conclusively the wide range of CFs in common between IBD and ID. Most importantly, the implication of the results is that strengthening the immune system against both infection and autoimmune diseases requires the discipline to 1) remove exposure to a broad range of toxic substances and 2) eliminate toxic behaviors.

Special mention should be made of cigarette smoking. It was identified as a candidate CF in the analysis, but was not included in Table 1 because of conflicting information relative to its UC impact and its COVID-19 impact. Because of its importance, however, it will be addressed in detail here.

#### 5.2.1 IBD impact of cigarette smoking

The association between cigarette smoking and IBD is complex. In our literature search, terms related to smoking, IBD, and COVID-19 returned 10−100-fold higher matches than other potential CFs. Cigarette smoking (both current and former use) remains the strongest environmental risk factor associated with developing CD in Western countries, and portends a more severe disease course compared to non-smokers [28]. Interestingly, current smoking is inversely related to developing UC in some studies, and smoking cessation has been associated with subsequently developing UC [29,30]. However, regular smoking that begins at a young age (10 years) is associated with a higher risk of developing UC [31]. Maternal smoking and passive smoke exposure in childhood is associated with developing CD [30,31].

Smoking likely influences intestinal inflammation through multiple mechanisms, given the numerous compounds found in tobacco, and individual genetic and epigenetic susceptibility [32,33]. Cigarette smoke can 1) negatively impact tight junctions and intestinal barrier function, allowing for increased intestinal permeability and translocation; 2) interfere with Paneth cell function, reducing antimicrobial peptide release and clearance of pathogens; 3) induce epithelial oxidative damage; and 4) contribute to dysbiosis [32].

Smoking also directly affects the immune system by modulating Th-1 response, clonal expansion of CD8+ and Th17 cells, and pro-inflammatory cytokine release [32]. Nicotine may have a positive impact on mucus production and adhesion molecule expression, though clinical trials with smoking and nicotine have had mixed results without a clear benefit in the treatment of UC due to side effects [[32], [33], [34], [35]]. It is well-established that smoking contributes to lung dysfunction, and a recent meta-analysis confirms that smoking is associated with the severe COVID-19 disease progression [36].

#### 5.2.2 COVID-19 impact of cigarette smoking

To date, the available information is somewhat controversial. The World Health Organization (WHO) recently confirmed that smokers are at greater risk of severe complications following SARS-CoV-2 virus infection, compared to non-smokers [37].

On the other hand, the known anti-inflammatory properties of nicotine have been suggested as a possible explanation for the low smoking prevalence in hospitalized patients in China [38].

### 5.3 CFs, inflammatory bowel disease, and SARS-CoV-2 infection

Among the many contributing factors that are common to ID and IBD, and that can be potentiated by the recently emerged SARS-CoV-2 infection, three factors appear to be of prominent and widespread relevance: advanced glycation end products (AGEs), high-fat diet (HFD), and Vitamin D Deficiency. *De facto*, these three CFs exert their risk effects on the entire world population.

AGEs can enter our organism exogenously from foods, especially foods heated to high temperatures. Additionally, AGEs can be formed endogenously; the non-enzymatic glycation reaction between sugars and proteins starts in our organism from the very first hours of life, independently of any (epi)genetic factor and obeying only the Guldberg and Waage's law of mass action.

Germane observations hold for HFD and Vitamin D Deficiency: they can be restored to normal healthy values through regulating their input amounts or, restating, by controlling our feeding behavior. Obviously, these considerations do not apply to CFs such as Renal Transplantation or Rituximab.

Moreover, and equally relevant, AGEs, HFD, and Vitamin D Deficiency are noteworthy since, as mentioned above, they converge with SARS-CoV-2 infection into an inflammatory picture dominated by alterations of the inflammasome-components. More specifically, the inflammatory context dominated by NLRP3 inflammasome activation underlies and links together CFs and IBD, is shared with SARS-CoV-2 infection as well, and emerges from the data below.

AGEs induce/upregulate/exacerbate the expression of the Receptor of Advanced Glycation End products (RAGE) [[39], [40], [41], [42], [43]]. By binding to RAGE on the cell surface, AGEs trigger the release of pro-inflammatory tumor necrosis factor-alpha (TNF-α) [44], the pro-inflammatory cytokine that has a pathological role in IBD [45,46]. Moreover, TNF-α sustains the progression of IBD by promoting the expression of the pro-inflammatory interleukins IL-1β, IL-6, and IL-33 [46,47]. RAGE can also bind S100A8 and S100A9, two proteins predominantly found as calprotectin (S100A8/A9) and involved in inflammatory processes and immune response [48]. Binding of calprotectin (S100A8/A9) to RAGE results in significantly increased secretion of pro-inflammatory interleukin (IL)-6, IL-8, IL-1β, and TNF-α [49], thus leading to a further amplification of the pro-inflammatory cascade. Moreover, in a positive feedback loop, the AGEs-induced/upregulated RAGE can mediate gene expression of S100A8 and S100A9 [50].

Calprotectin is involved in the regulation of the NLRP3 inflammasome [51] that is directly implicated in IBD [52], and is one of the most useful tools for monitoring intestinal inflammation for clinical management of IBD [53].

The disintegrated immunological scenario that links AGEs to IBD also characterizes the relationship between High Fat Diets (HFDs) and IBD. HFDs have been repeatedly related to IBD [54,55], most possibly because of the fatty acids capacity of inducing a systemic chronic low-grade inflammation [56] marked by elevated production of the pro-inflammatory cytokines interleukin (IL)-1β [57,58], IL-6 [59], and TNF-α [60] in the gut. The molecular basis underlying the pathological connection between HFDs and IBD appears to be the NLRP3 inflammasome [61], with saturated fatty acids promoting NLRP3 inflammasome activation [62] and unsaturated fatty acids impeding NLRP3 activity [62,63].

Vitamin D deficiency has been reported to occur frequently in people with IBD [[64], [65], [66]]. Again, NLRP3 inflammasome activation appears to play a main role in the connection between Vitamin D deficiency and IBD. Indeed, Vitamin D exerts potent anti-inflammatory effects [67] by enhancing the Vitamin D Receptor signaling that inhibits NLRP3 inflammasome activation and IL-1β secretion [68].

On the whole, a molecular picture emerges that correlates different CFs to the etiology and chronicization of IBD. In addition, these CFs also relate to SARS-CoV-2 infection. In fact, as in IBD, the inflammation that characterizes COVID-19 is dominated by high cytokine levels (IL-2R, IL-6, IL-10, and TNF-α) [69]. Moreover, it has been proposed that RAGE and its ligands may play a pivotal role in COVID-19 pneumonia [70,71], particularly in the presence of concomitant comorbidities like diabetes mellitus [72]. Clinically, data show that elevated calprotectin levels discriminate severe from mild COVID-19 [73] and correlate with inferior clinical outcomes [74].

Of note, SARS-CoV ORF8b triggers NLRP3 inflammasome activation and IL-1β release by binding to the leucine rich repeat (LRR) domain of NLRP3, resulting in macrophage pyroptosis [75,76]. Accordingly, inflammasome activation has been found in COVID-19 patients with cardiac involvement [77] and in the lungs of patients with fatal COVID-19 [78].

In sum, IBD and COVID-19 share CFs able to induce immune dysfunctional disorders that have a minimum common denominator in NLRP3 inflammasome activation. Such commonality leads to theoretical predictions of an increase of IBD morbidity as a sequel of the current SARS-CoV-2 pandemic, particularly in those patients with IBD who have active inflammation.

## 6 New paradigm required for preventing and treating infectious and chronic diseases

We have presented the foundation for a unified theory of chronic-infectious disease from the perspectives of causation and prevention. As a result, our findings suggest a need for paradigmatic shift in medical approaches to disease. The current approach to both infectious and chronic disease in Western medicine is often external-treatment-based (*i.e.*, providing a drug, vaccine, radiation, surgery, *etc.*) to reduce symptoms without sufficiently addressing the underlying modifiable factors that enabled the disease to emerge. This study highlights modifiable factors (toxic exposures and behaviors) that contribute to disease pathogenesis via various mechanisms of immune dysfunction, and shows commonality between IBD and COVID-19. Eliminating these factors as comprehensively and rapidly as possible is prudent, and should be pursued in parallel with treatment.

## Author contributions

Kostoff RN contributed to this paper with conception, data analysis, and writing the manuscript; Briggs MB participated in data analysis, results validation, and table development; Kanduc D participated in data analysis and writing the manuscript; Shores DR contributed to query development, background development, and editing; Kovatsi L, Vardavas A, and Porter AL contributed to writing the manuscript and editing; all the authors approved the final version of the manuscript.

## Declaration of Competing Interest

The authors declare that they have no known competing financial interests or personal relationships that could have appeared to influence the work reported in this paper.

## Figures and Tables

**Table B1:** Biomarkers that link contributing factors to IBD and immune degradation/COVID-19.

Contributing Factor	Cat.	Dis.	Biomarkers	Ref.
Advanced glycation end products	1	IBD	Carboxymethyllysine; enzymatic antioxidative pathways; IL‐6; inflammation; inflammatory cytokine levels; inflammatory response; MDA; Oxidative stress; SOD; TNF‐α	[79]
		ID	Caspase-1; IL-1; immunosuppressive; innate immune responses; M1 polarization of macrophages; macrophages; NLRP3 inflammasome; pro-inflammatory cytokines in BMDMs; RNA virus infection; type-I interferon	[80]
		COV	Accumulation of advanced glycation end products; acute respiratory distress syndrome; atherothrombotic state; chronic inflammatory; cytokine storm; degrade tissue debris; exaggerated cytokine response; diabetes; impaired immune response; inflammatory response; increased metabolic activity; multi-organ failure; obesity; septic shock; tissue damage; viral infection	[81]
Alcoholism	1	IBD	Crohn Disease (CD); inflammatory bowel disease (IBD); ulcerative colitis (UC)	[82]
		ID	Alveolar macrophage function; alveolar macrophage immune dysfunction; granulocyte-macrophage colony-stimulating factor receptor; immune dysfunction; intracellular zinc; phagocytic function; zinc bioavailability; zinc deficiency; zinc metabolism	[83]
		COV	Lung fibrosis; pulmonary fibrosis	[84]
Carrageenan	1	IBD	EGR-1; IL-8; intestinal epithelial barrier integrity; Intestinal inflammation; NF-κB; pro-inflammatory transcription factors; UC; zonula occludens	[85]
		ID	Ccl2; Ccl7; Cxcl1; Cxcl2; inflammatory secretome; leukocyte; macrophages; multiple collagens; neutrophils; peripheral inflammation; tissue inflammation	[86]
Consumer antimicrobials	1	IBD	Colon cancer; colon tumorigenesis; colonic inflammation; IBD; intestinal barrier function; TLR4 signaling	[87]
		ID	Colon cancer; colon tumorigenesis; colonic inflammation; IBD; intestinal barrier function; TLR4 signaling	[87]
High-fat diet	1	IBD	Cholesterol; colitis; colonic epithelial cells; colonic inflammation; free fatty acids; gp91; IBD; lamina propria cells; MLCK tight junction; mucosal barrier permeability; mucosal oxidative stress; mucosal pro-inflammatory cytokines; ROS; triglyceride	[88]
		ID	Gut-associated lymphoid tissue; IBD; lamina propria lymphocytes; lipotoxicity; small intestinal intraepithelial lymphocytes	[89]
		COV	Endotoxemia; gut microbiome dysbiosis; immunosilent/immunoinhibitory bacteroidetes LPS; intestinal permeability; lipopolysaccharide (LPS); oxidative stress; proinflammatory LPS; TLR4	[90]
High-salt diet	1	IBD	activated peritoneal macrophages; CD11b + macrophages; CD3+CD4+CD25+Foxp3+ T cells; CD4+IFN-γ+IL-17+ T cells; colitis; IFN-γ; IL-1; IL-10; IL-6; lamina propria; lamina propria mononuclear cells; macrophages; mesenteric lymph node; mouse inducible nitric oxide synthase; p38 phosphorylation; p38/MAPK axis; peritoneal macrophage inflammation; proinflammatory; SGK1; spleen; TGF-β; Th1; Tregs	[91]
		ID	Autoimmune disease; inflammatory disease; inflammatory kidney disease; innate and adaptive immune systems; innate immune system components	[92]
Malnutrition	1	IBD	Catalase (CAT); colon epithelium; IL-1β; IL-4; inflammatory mediators; lipoperoxidation; NO; oxidative stress; reduced (GSH) and oxidized (GSSG) glutathione; TNF-α; total superoxide dismutase (tSOD)	[93]
		ID	chronic inflammation; disordered nutrient assimilation; immune defect; immune dysfunction; immunopigenome; infections	[94]
		COV	cytokine storm; high adiposity; infection; low lean mass; proinflammatory cytokines	[95]
Maternal smoking	1	IBD	cg25189904 in GNG12 gene; DNA methylation	[96]
		ID	Asthma; cotinine; respiratory infections; toll-like-receptor (TLR)	[97]
		COV	COVID-19	[98]
Nitrosamines	1	IBD	chronic inflammation; hydrogen peroxide; mutagenesis; nitrosation; N-nitrosamine derivatives; NO; oxidation; ROS; secondary amines; superoxide; UC	[99]
		ID	Arachidonic acid metabolites; IgG; leukotrienes; N-nitrosamines (N-nitrosodimethylamine and N-nitrosodiethylamine); oncosuppression; p53 transcription factor	[100]
Polysorbate-80	1	IBD	Endoplasmic reticulum stress; gut homeostasis; IL-10; immune-inflammatory disorders; IBD; inflammatory responses; intestinal goblet cells; TLR5	[101]
		ID	sensitization rates	[102]
Refined carbohydrates	1	IBD	CD; DNA methylation; histone modification; IBD; miRNAS; UC	[103]
		ID	Adaptive immunity; chronic inflammation; impaired host defense; innate immune system; peripheral inflammation	[104]
		COV	Chronic inflammation; COVID-19; dementia; impairs adaptive immunity; impaired host defense; innate immune system; neurodegenerative disease; neuroinflammatory; type 2 diabetes; obesity; peripheral inflammation	[104]
Sedentary	1	IBD	Adipokines; colonic blood flow (CBF); colonic microcirculation; COX-2; disease activity index; GPx mRNAs; hypoxia inducible factor (HIF)-1α; IFNγ; IL-10; IL-17; IL-1β; IL-6; iNOS; intestinal microcirculation; MDA; myokines; oxidative stress; reduced glutathione (GSH); SOD-1; SOD-2; SOD; TNF-α; weight	[105]
		ID	Atherosclerosis; cytokines; development of tumors; immune cells with pro-inflammatory characteristics in adipose tissue; insulin resistance; neurodegeneration	[106]
		COV	Body mass index; obesity; severe infection	[107]
Vitamin D deficiency	1	IBD	Anti-inflammatory; autoimmune diseases; cathelicidin; DEFB4 (defensin, beta 4); gastrointestinal inflammation; gut microbiome	[108]
		ID	Acetylcholine; actinobacteria; autoimmune arthritis; bacteroidetes; pantothenic acid deficiency; bowel symptoms; cortisol; firmicutes; pain; proteobacteria; sleep; vitamin D blood levels	[109]
		COV	cytokine storm; dengue fever; innate immune system; virus replication; vitamin D deficiency	[110]
Antibiotics	2	IBD	CD; IBD; UC	[111]
		ID	Adaptive immunity; immune responses; infection; microbiota homeostasis	[112]
Cisplatin	2	IBD	Anorexia; bloody stool; colitis; diarrhea; UC	[113]
		ID	Humoral immunity; immunotoxicity; leukocytic count; lymphocyte percentage; lymphoid tissue; mycoplasma gallisepticum; neutrophil percentage; phagocytic activity; total protein; γ-globulin concentration	[114]
Opioids/Morphine	2	IBD	ATP release; ATP-induced currents; bacterial translocation; colonic inflammation; connexin43 (Cx43); enteric glia; IL-1β mRNA; IL-6; leaky gut; microbial dysbiosis; P2X currents; P2 × 4; P2 × 7; purinergic P2X receptor activity	[115]
		ID	Acetylcholinesterase; AP-1; bone marrow-derived macrophages; CREB; innate immunity; microglia; microRNA-124; NF-κB p65; pain; phospho-p65; TLRs; TNFR-associated factor 6	[116]
		COV	COVID-19	[117]
Oral contraceptives	2	IBD	UC	[118]
		ID	Autoimmune diseases; blood urea nitrogen; cytokines; glomerular immune complex deposition; immune system; kidney disease; proteinuria; splenic leukocytes; TLR7/9 cytokine production	[119]
Radiotherapy	2	IBD	Bloating/distension; chronic gastrointestinal symptoms; fecal incontinence; flatulence; frequency; loose stool; rectal bleeding; urgency	[120]
		ID	Blood proteome; inflammatory responses	[121]
		COV	Cancer; carcinoma; COVID-19	[122]
Renal transplantation	2	IBD	Diarrhea; diverticular colon disease; fecal occult blood; gastrointestinal pathologies; HBoV; HBoV-3; immunosuppressed; RVA; RVA G3	[123]
		ID	Diarrhea; Rotavirus A (RVA) and Human Bocavirus (HBoV) in fecal samples	[124]
		COV	Chronic kidney disease; infection	[125]
Rituximab	2	IBD	Autoimmune disease; CD20 + lymphocytes; CD; granulomas; granulomas; ileocolitis; inflammation; inflammation; ulceration; ulceration; UC	[126]
		ID	Hypogammaglobulinemia; IgG; severe infections	[127]
		COV	Bilateral pneumonia; respiratory insufficiency; rheumatic and musculoskeletal disease; rheumatoid arthritis; SAR-CoV-2 infection; Sjögren syndrome; systemic lupus erythematosus; systemic vasculitis	[128]
Vaccines	2	IBD	IBD	[129]
		ID	Non-influenza infections	[130]
Cytomegalovirus	3	IBD	Autoantibodies; autoimmune; CD13 (aminopeptidase N); CD13 (aminopeptidase N) specific autoantibodies; human cytomegalovirus infection	[131]
		ID	Autoimmune diseases; HCV; immune dysfunction; immunosuppression; inflammation	[132]
		COV	Cytomegalovirus (CMV); herpesvirus infection; immune senescence; infection; metabolic disorders	[133]
N-ethyl-N-nitrosourea	3	IBD	Apoptosis; chronic diarrhoea; endoplasmic reticulum stress; IFN-γ; IFN-γ; IL-13; IL-1β; inflammation; intestinal mucus barrier; intestinal goblet cells; intestinal permeability; leukocytes; MUC2 mucin; Th1 cytokines; Th2 cytokines; TNF-α; ulcerative colitis-like phenotype; unfolded protein response	[134]
		ID	Cell surface receptors; innate immunity; toll-like receptors	[135]
Trichothecenes	3	IBD	Caco-2 cell monolayer; celiac disease; chronic IBD; claudin protein levels; claudins; colonic epithelial barrier; FITC-dextran; ZO-1	[136]
		ID	B cells; chemokines; cytokines; double-stranded RNA-(dsRNA)-activated protein kinase; hematopoetic cell kinase (Hck); immune suppression; immune system; inflammatory genes; leukocyte apoptosis; macrophages; MAPKs; T-cells	[137]
Zearalenone	3	IBD	Autoimmune; IFN-γ; IgE; IL-2; infectious diseases; macrophages; monocytes; proinflammatory cytokines; Th1 cytokines; Th1/Th2 cell polarization; Th2 cytokines; ZEN concentrations	[138]
		ID	Autoimmune; IFN-γ; IgE; IL-2; infectious diseases; macrophages; monocytes; proinflammatory cytokines; Th1 cytokines; Th1/Th2 cell polarization; Th2 cytokines; ZEN concentrations	[138]
Air pollutants	4	IBD	Epithelial cells; immune activation; intestinal microbiota; systemic inflammation	[139]
		ID	IgG; IgM; monocytes; red blood cells; white blood cells	[140]
		COV	COVID-19	[141]
Aluminum	4	IBD	Colitis; colonic myeloperoxidase activity; epithelial cell renewal; granuloma formation; immune toxicity; inflammatory cytokines expression; intestinal barrier function; intestinal inflammation	[142]
		ID	ANAE(+) cells; complement 3 (C3) level; IL-2; immunotoxicity; splenic iron; splenic zinc; TNF-α	[143]
Benzene	4	IBD	Abdominal pain; bloody diarrhea; CD11b + cell infiltration; colonic migrating motor complex frequency and amplitude; cramping; epithelial barrier permeability; IL-1β concentrations; inflammation; innate immune activation; migrating CD11b + cells	[144]
		ID	ALA-D activity; CD80; CD86; IL-8; lymphocytes; monocytes	[145]
		COV	COVID-19	[146]
Benzo(a)pyrene	4	IBD	Colonic mucosa; gut microbiota dysbiosis; intestinal inflammation; lesions	[147]
		ID	IgM; lysosomal membrane stability; lysozyme activity; phagocytosis; total RBC and WBC	[148]
Bisphenol	4	IBD	IBDs; oral tolerance; pro-inflammatory cellular responses	[149]
		ID	Anti-E. coli IgG; immune response; inflammatory markers; intestinl barrier functions; Th1/Th17 inflammation	[150]
		COV	Angiotensin-converting enzyme 2; GPR30; infection; membrane-bound oestrogen receptor; nuclear oestrogen receptors; nuclear receptor oestrogen-related receptor gamma; transmembrane serine protease 2	[151]
Cadmium	4	IBD	Bacteria-induced cytokine responses; gastrointestinal autoimmune diseases; IBD; intestinal inflammation; transepithelial electric resistance	[152]
		ID	Apoptosis induction; glutathione; IgE-mediated histamine release; immunotoxic; innate immune system; macrophages; mast cells; nitrite production; oxidative stress; ROS; redox imbalance; TNF-α	[153]
		COV	Adaptive immunity; airway inflammation; apoptosis; barrier function; immunotoxicity; mucociliary clearance; oxidative stress	[154]
Chlorpyrifos	4	IBD	Chronic inflammation; colon weights; haptoglobin; immune tolerance; luminal IgG levels; neutrophils; Treg cells; spleen T cells; T-cells; Treg-related genes; TNF-α	[155]
		ID	Immune response; oxidative stress; ROS	[156]
Fluoridated water	4	IBD	Gut microbiota; intestinal inflammation	[157]
		ID	Catalase; cellular immunity; humoral immunity; immunotoxicity; MDA; monocytes; neutrophils; oxidative stress; peripheral blood lymphocytes; peroxidase; plasma IgG; spleen; splenocytes counts; thymus	[158]
Mercury	4	IBD	UC	[159]
		ID	Humoral immune response; IgE	[160]
		COV	Airway inflammation; apoptosis; immunotoxicity; impaired mucociliary clearance; influenza; oxidative stress; reduced barrier function; respiratory diseases (COPD, bronchitis); respiratory dysfunction; respiratory syncytial virus; viral diseases	[154]
Microplastics	4	IBD	Dysbiosis; gut epithelium; gut's epithelial permeability; immune cell toxicity; immune response; inflammatory intestinal balance; intestinal homeostasis; microbiota	[161]
		ID	Dysbiosis; gut epithelium; gut's epithelial permeability; immune cell toxicity; immune response; inflammatory intestinal balance; intestinal homeostasis; microbiota	[161]
Nickel	4	IBD	Metal hypersensitivity; T-cells; ulcerative lesions	[162]
		ID	Apoptosis; cytokines; development of immune organs; immunoglobulins; immunotoxicity; immunotoxicological; inflammation; inflammation; lymphocyte; macrophages; natural killer cells; nuclear factor-κB; MAPK pathways; T and B lymphocytes; toll like (TL)4	[163]
Paraquat	4	IBD	IBD; sulfide; ulcerative colitis	[164]
		ID	Apoptosis; apoptosis of lymphocytes; immunotoxicity; memory cells; memory immune response; number of effector and memory lymphocytes; primary immune response; secondary immune response; serum total IgG; serum anti-KLH IgG and KLH-responsive CD4 T cells and B cells	[165]
PCB (polychlorinated biphenyls)	4	IBD	Catalase; free radical production; GSH-Px; high-molecular-weight antioxidants; low-molecular-weight antioxidants; lymphocytes; neutrophils; oxidative injuries; SOD; vitamin A; vitamin C; vitamin E	[166]
		ID	Leukocyte phagocytosis	[167]
PFOA (perfluoroctanoic acid)	4	IBD	CD; PFAS serum levels; PFOS serum levels; UC	[168]
		ID	IL-1β; IL-21; Myd88/NF-κB pathway; myeloid differentiation 88; P65 transcription factor; pro-inflammatory cytokine IL-1β; spleen; TLR2	[169]
		COV	Immunotoxic; PFAS concentrations; perfluorobutanoic acid (PFBA); short-chain PFBA	[170]
Polycyclic aromatic hydrocarbons	4	IBD	Abnormal cell growth; barrette's esophagus; blood circulation; cell dysfunction; chronic inflammation; CDs; DNA adducts; gene mutation; neural regulation; ulcer formation; SOD; UC	[171]
		ID	Antibody-forming cell response; immunosuppression; immunotoxicity	[172]
		COV	Adverse respiratory effects; COVID-19; severe respiratory infections	[173]
Silica dust	4	IBD	CD; UC	[174]
		ID	autophagy; Bcl-2; Beclin1; macrophages; NR8383 cells; PIK3C3	[175]
		COV	HIV; Interstitial lung disease; Silicosis; tuberculosis (TB)	[176]
Sodium dichromate dihydrate	4	IBD	Bile acid concentrations; body weights; epithelial hyperplasia; erythrocyte counts; focal ulceration; glycogen depletion; histiocytic cellular infiltration; mean red cell volumes; microcytic hypochromic anemia; nonneoplastic lesions; red cell hemoglobin values; serum cholesterol; triglyceride; urine specific gravity; urine volume	[177]
		ID	Alveolar macrophages; antibody responses; humoral immune system; phagocytic activities	[178]
		COV	Arsenic; cadmium; chromium; copper; creatinine; inflammatory; ferritin; lead; manganese; mercury; neutrophil count; selenium; serum cytokines (IL-1B, IL-2R, IL-6, IL-8, IL-10, TNFα); thallium; toxic metals; trace metals; urinary creatinine-adjusted copper; white blood cell count	[179]
Titanium dioxide	4	IBD	Body mass; dysbiosis; GI microbiome; immune response; inflammation; inflammatory cytokines; mucin expression; mucosal barrier; mucus production	[180]
		ID	Immunomodulation; immunosuppressant; splenocytes proliferation; T-cell; Th1 type cytokines (IL-2, interferon-γ)	[181]
Triclocarban	4	IBD	Colitis; gut microbiota	[182]
		ID	Cxcl-clc; IL-1β; IL-4; IL-8; immune cell recruiting; irak4; lipid peroxidation; myd88; NF-κB; NO; NO synthase gene; oxidative damage; oxidative stress; SOD; TNF-α; total antioxidant capacity expression; traf6; trif	[183]
Turpentine	4	IBD	Colonic distension; NMDA receptor; visceral pain	[184]
		ID	Concanavalin A; IgG; inflammation; IL-6	[185]
Low socioeconomic status	5	IBD	Autoimmune disorders; CD	[186]
		ID	Adverse events following immunization	[187]
		COV	COVID-19	[188]
Posttraumatic stress	5	IBD	Autoimmune disorders; immune abnormalities; rheumatoid arthritis	[189]
		ID	Dysregulation of genes; innate immune; NF-κB	[190]
Psychological stress	5	IBD	Intestinal permeability; mast cells activation; salivary cortisol	[191]
		ID	Cytokines; IFN-γ; IL-1β; IL-10; IL-6; immune cell populations; immune function; macrophage; Th1 cell-mediated cellular immunity; Th2 cell-mediated humoral immunity and macrophage activities; Th1 cell; Th2 cell	[192]
		COV	Cytokine storm; diabetes; glucocorticoid insufficiency; hyperinflammation; hyperinflammatory responses; hypertension; immune dysregulation; inflammation; IL-6 hypersecretion; lung conditions; obesity	[193]
Restraint stress	5	IBD	Colonic mucin glycoprotein; goblet cells; mast cell counts; mucin; myeloperoxidase content	[194]
		ID	CD4+ T lymphocytes; CD8 + T lymphocytes; cytokine levels; IL-10; IL-17; IL-6; immunosuppression; splenic natural killer cell activity; splenocyte proliferation	[195]
Sexual abuse	5	IBD	CD; IBD; Irritable bowel syndrome; UC	[196]
		ID	C-reactive protein; IL-6; inflammatory activity; inflammatory responses; TNF-α	[197]

## Appendix A

### Vitamin D Deficiency

IBD: “Vitamin D deficiency leads to dysbiosis of gut microbiome and reported to cause severe colitis.” [108]

ID: “Vitamin D deficiency changes the intestinal microbiome reducing B vitamin production in the gut. The resulting lack of pantothenic acid adversely affects the immune system, producing a "pro-inflammatory" state associated with atherosclerosis and autoimmunity” [109]

COV: “It is already evident that the prevalence of vitamin D deficiency in Europe, particularly in the northern mid-latitudes, seems to be closely aligned to increased COVID-19 morbidity and mortality” [110]

#### CATEGORY 2 - IATROGENIC

##### Antibiotics

IBD: “Higher cumulative exposure to systemic antibiotic therapy, particularly treatments with greater spectrum of microbial coverage, may be associated with a greater risk of new-onset IBD and its subtypes……suggest the need to further emphasise antibiotic stewardship to prevent the rise in dysbiosis-related chronic diseases, including IBD.” [111]

ID: “infants, who are subjected to frequent antibiotic exposures due to their vulnerability to infection, reflect increased susceptibility to a wide spectrum of diseases, including infection, in later life. Antibiotics induce perturbations of the microbiota or dysbiosis, which in turn alters the host immune responses against pathogens” [112]

##### Cisplatin

IBD: “He was diagnosed with lung adenocarcinoma…..After two cycles of cisplatin and vinorelbine administration, he experienced persistent diarrhoea [sic] and anorexia. Findings of the colonoscopy revealed a pancolitis type of UC” [113]

ID: “Cisplatin induced marked inhibition of cellular immunity as exhibited by significant decrease of leukocytic count, lymphocyte percentage and phagocytic activity with marked increase in neutrophil percentage. Humoral immunity represented by marked inhibition in total protein and γ-globulin concentration and significant inhibition in antibody titer against *Mycoplasma gallisepticum* were recorded” [114]

##### Opioids/Morphine

IBD: “Prolonged morphine treatment results in a "leaky" gut, predisposing to colonic inflammation that is facilitated by microbial dysbiosis and associated bacterial translocation.” [115]

ID: “we show that morphine suppresses the innate immunity in microglia and bone marrow-derived macrophages through differential regulation of TLRs and acetylcholinesterase. Either morphine or inhibition of acetylcholine significantly promotes upregulation of microRNA-124 (miR-124) in microglia, bone marrow-derived macrophages, and the mouse brain, where miR-124 mediates morphine inhibition of the innate immunity by directly targeting a subunit of NF-κB p65 and TNFR-associated factor 6 (TRAF6).” [116]

COV: “Substance use disorders can also increase the risk of adverse COVID-19 outcomes through immunosuppression…..For example, opioids exert various suppressive effects on both the innate and adaptive immune systems, especially among those who have long used substances (> = 90 days in a year). Largely by binding to the mu receptor and modulating various downstream cellular signaling pathways, opioids impair the recruitment and function of virtually all immune cells, such as macrophages, NK cells, granulocytes, and B and T lymphocytes” [117]

##### Oral Contraceptives

IBD: “This study provides evidence of an association between the use of oral contraceptives and the onset risk of UC” [118]

ID: “17α-ethinyl estradiol (EE), a synthetic analog of 17β-estradiol, is prescribed commonly and found in oral contraceptives and hormone replacement therapies. Surprisingly, few studies have investigated the immunoregulatory effects of exposure to EE, especially in autoimmunity….. EE-exposed mice had increased proteinuria as early as 7 weeks of age. Proteinuria, blood urea nitrogen, and glomerular immune complex deposition were also exacerbated when compared to controls. Production of cytokines by splenic leukocytes were altered in EE-exposed mice. Our study shows that oral exposure to EE, even at a very low dose, can exacerbate azotemia, increase clinical markers of renal disease, enhance glomerular immune complex deposition, and modulate TLR7/9 cytokine production in female MRL/lprmice” [119]

##### Radiotherapy

IBD: “Fifty percent of patients develop chronic gastrointestinal (GI) symptoms following pelvic radiotherapy that adversely affect quality of life.” [120]

ID: “Systemic responses to radiation detected at the blood proteome and metabolome levels are primarily related to the intensity of radiation-induced toxicity, including inflammatory responses” [121]

COV: “The results of the current study demonstrated a possible association between recent receipt of oncologic treatment and a higher risk of death among patients with carcinoma who are hospitalized with COVID-19.” [122]

##### Renal Transplantation

IBD: “patients after kidney transplantation have significant risk of gastrointestinal pathologies and require detailed diagnostic endoscopy” [123]

ID: “Immunosuppressive therapy causes severe impairment of host defense and diarrhea is a frequent complication in renal transplant recipients.” [124]

COV: “Chronic kidney disease (CKD) and immunosuppression, such as in renal transplantation (RT), stand as one of the established potential risk factors for severe coronavirus disease 2019 (COVID-19). Case morbidity and mortality rates for any type of infection have always been much higher in CKD, haemodialysis (HD) and RT patients than in the general population.” [125]

##### Rituximab

IBD: “There are reports of rituximab-associated ulcerative colitis; however, we report for the first time, two cases of rituximab-induced Crohn's disease in elderly patients treated for lymphoma.” [126]

ID: “Following rituximab administration, worsening hypogammaglobulinemia was noted. There was an increase in severe infections after rituximab use in the study cohort” [127]

COV: “High rates of severe disease and death due to SARS-CoV-2 infection in rheumatic disease patients treated with rituximab: a descriptive study” [128]

##### Vaccines

IBD: “receiving seven vaccines or more during childhood increased the risk of developing inflammatory bowel disease by nine-fold (odds ratio = 9.2, confidence interval = 2.9-29.4)” [129]

ID: “We randomized 115 children to trivalent inactivated influenza vaccine (TIV) or placebo. Over the following 9 months, TIV recipients had an increased risk of virologically-confirmed non-influenza infections (relative risk: 4.40; 95 % confidence interval: 1.31–14.8). Being protected against influenza, TIV recipients may lack temporary non-specific immunity that protected against other respiratory viruses.” [130]

#### CATEGORY 3 – BIOTOXINS

##### Cytomegalovirus

IBD: “Cytotoxic CD13-specific autoantibodies were identified in 66 % of the sera obtained from HCMV-IgG positive patients with ulcerative colitis and in 58 % of the sera obtained from HCMV-IgG positive patients with Crohn's disease, but not in control individuals. These cytotoxic autoantibodies may interfere with biological cell functions and could thereby contribute to the chronic inflammation in patients with IBD.” [131]

ID: “During active infection, CMV modulates host immunity, and CMV-infected patients often develop signs of immune dysfunction, such as immunosuppression and autoimmune phenomena.” [132]

COV: “Cytomegalovirus (CMV), a persistent herpesvirus infection whose prevalence increases with age, is a major modulator of immune function and several observations suggest that infection might act to influence clinical outcome following SARS-CoV-2 infection.” [133]

##### N-Ethyl-N-Nitrosourea

IBD: “By murine N-ethyl-N-nitrosourea mutagenesis we identified two distinct noncomplementing missense mutations in Muc2 causing an ulcerative colitis-like phenotype. … Mutant mice showed aberrant Muc2 biosynthesis, less stored mucin in goblet cells, a diminished mucus barrier, and increased susceptibility to colitis induced by a luminal toxin. Enhanced local production of IL-1beta, TNF-alpha, and IFN-gamma was seen in the distal colon, and intestinal permeability increased 2-fold. The number of leukocytes within mesenteric lymph nodes increased 5-fold and leukocytes cultured in vitro produced more Th1 and Th2 cytokines (IFN-gamma, TNF-alpha, and IL-13).” [134]

ID: “N-Ethyl-N-nitrosourea (ENU) mutagenesis is an efficient tool for the creation of aberrant monogenic innate immune response phenotypes.” [135]

##### Trichothecenes

IBD: “trichothecene deoxynivalenol (DON), a fungal metabolite found in grain-based human diets, … demonstrate that DON disintegrates a human Caco-2 cell monolayer … followed by a decrease in transepithelial resistance and an increased permeability of marker molecules, such as lucifer yellow and FITC-labeled dextran. … increase in paracellular transport of FITC-dextran is demonstrated in vivo in B6C3F1 mice … In vitro claudin protein levels are decreased and correlated with a displacement within the cells in vitro and in vivo, accompanied by a compensatory up-regulation of mRNA levels of claudins and their binding partner ZO-1.” [136]

ID:“Macrophages, T cells, and B cells of the immune system are central targets of deoxynivalenol (DON) and other trichothecenes-mycotoxins that can be immunostimulatory or immunosuppressive depending on dose, exposure frequency and timing of functional immune assay.” [137]

##### Zearalenone

IBD:“Changes in Th1 and Th2 cytokine concentrations in ileal Peyer's patches in gilts exposed to zearalenone” [138]

ID:“Changes in Th1 and Th2 cytokine concentrations in ileal Peyer's patches in gilts exposed to zearalenone” [138]

#### CATEGORY 4 – OCCUPATIONAL/ENVIRONMENTAL

##### Air Pollutants

IBD: “Exposure of the bowel to air pollutants occurs via mucociliary clearance of PM from the lungs as well as ingestion via food and water sources. Gaseous pollutants may also induce systemic effects. Plausible mechanisms mediating the effects of air pollutants on the bowel could include direct effects on epithelial cells, systemic inflammation and immune activation, and modulation of the intestinal microbiota.” [139]

ID: “Numerous immune biomarkers were significantly elevated in these sensitive children, compared with normal children, and several biomarker alterations in these children were related to high concentrations of air pollutants in the home. The strongest and most significant associations were seen between high indoor nitrogen dioxide concentrations and increased white blood cells, monocytes, red blood cells, and immunoglobulin G (IgG), as well as decreased immunoglobulin M (IgM) and Klebsiella pneumoniae-specific IgM. Bacterial-specific IgGs were related significantly to formaldehyde concentrations. These findings suggest the important role of indoor air pollutants in immune reactions.” [140]

COV: “Even within a single city, higher levels of air pollution are associated with an adverse impact on COVID-19 risk.” [141]

##### Aluminum

IBD: “Aluminum increased the intensity and duration of macroscopic and histologic inflammation, colonic myeloperoxidase activity, inflammatory cytokines expression, and decreased the epithelial cell renewal compared with control animals. Under basal conditions, aluminum impaired intestinal barrier function. In vitro, aluminum induced granuloma formation and synergized with lipopolysaccharide to stimulate inflammatory cytokines expression by epithelial cells. Deleterious effects of aluminum on intestinal inflammation and mucosal repair strongly suggest that aluminum might be an environmental IBD risk factor.” [142]

ID: “studies in the literature showed that Al decreased splenic iron (Fe) and zinc (Zn) levels, but the effects of Al on splenic copper (Cu) level was ambiguous and controversial. Al exposure inhibited levels of ANAE(+) cells, the production of interleukin (IL)-2 and the functions of macrophages. With respect to other key cytokines, studies showed that Al suppressed the production of tumor necrosis factor (TNF)-α in vitro; effects of Al on TNF-α formation in vivo were less overt. Al exposure reduced complement 3 (C3) level,” [143]

##### Benzene

IBD: “TNBS colitis increased epithelial barrier permeability in-vitro and in-vivo. Colonic IL-1β concentrations, colonic and systemic CD11b + cell infiltration, and the number of migrating CD11b + cells on colonic blood vessels were all increased in TNBS treated mice relative to controls. CMMC frequency and amplitude were inhibited in the distal and mid colon of TNBS treated mice.” [144]

ID: “reduced ALA-D activity, decreased CD80 and CD86 expression in monocytes and increased IL-8 levels were found in the GSA group compared to the control subjects. Furthermore, according to multiple linear regression analysis, benzene exposure was associated to a decrease in CD80 and CD86 expression in monocytes.” [145]

COV: “The airborne benzene concentration was the leading risk factor in 24% of the counties” [146]

##### Benzo(A)Pyrene

IBD:“BaP oral exposure significantly altered the composition and the abundance of the gut microbiota and led to moderate inflammation in ileal and colonic mucosa.” [147]

ID:“Exposure of fish to BaP resulted in a significant decrease of total RBC and WBC, lysozyme activity, lysosomal membrane stability, IgM level and antibacterial activity after 4 days and phagocytosis after 7 days of the experiment (P < 0.05). Totally, the results revealed BaP ability to suppress the fish immune function.” [148]

##### Bisphenol

IBD: “Oral exposure to bisphenols induced food intolerance and colitis in vivo by modulating immune response in adult mice” [149]

ID: “Perinatal oral exposure to low doses of bisphenol A, S or F impairs immune functions at intestinal and systemic levels in female offspring mice” [150]

COV: “this paper focuses on the potential role of BPA in promoting comorbidities associated with severe COVID-19, as well as on potential BPA-induced effects on key SARS-CoV-2 infection mediators, such as angiotensin-converting enzyme 2 (ACE2) and transmembrane serine protease 2 (TMPRSS2)…..BPA exposure may impact on the local expression of these SARS-CoV-2 infection mediators. Overall, the potential role of BPA on the risk and severity of COVID-19 merits further investigation.” [151]

##### Cadmium

IBD: “In human cell-based models, exposure to Cd and Pb is associated with reduced transepithelial electric resistance and changes in bacteria-induced cytokine responses. Although 1- and 6-week exposures did not have clear effects on the response to Salmonella infectious challenges, 1-week short-term treatments with CdCl2 tended to enhance intestinal inflammation in mice.” [152]

ID: “Functional endpoints tumour necrosis factor-alpha and nitrite production in lipopolysaccharide-activated macrophages were increased by cadmium exposure. In contrast, mast cell lipopolysaccharide-induced tumour necrosis factor-alpha and IgE-mediated histamine release were reduced by cadmium. These data indicate potentially differential effects of cadmium among murine innate immune cell types, where mast cells would be more susceptible to oxidative stress and their function might be at a higher risk to be modulated compared to macrophages.” [153]

COV: “The existing data demonstrate that As, Cd, Hg, and Pb exposure is associated with respiratory dysfunction and respiratory diseases (COPD, bronchitis). These observations corroborate laboratory findings on the role of heavy metal exposure in impaired mucociliary clearance, reduced barrier function, airway inflammation, oxidative stress, and apoptosis. The association between heavy metal exposure and severity of viral diseases, including influenza and respiratory syncytial virus has been also demonstrated. The latter may be considered a consequence of adverse effects of metal exposure on adaptive immunity. Therefore, reduction of toxic metal exposure may be considered as a potential tool for reducing susceptibility and severity of viral diseases affecting the respiratory system, including COVID-19.” [154]

##### Chlorpyrifos

IBD: “dietary exposure to CPF aggravated tissue injuries in mice with DSS-induced chronic colitis by suppressing T-cell populations and Treg polarization.” [155]

ID: “CPF-induced ROS regulates immune response by stimulating the antigen-presenting ability of head kidney in carp.” [156]

##### Fluoridated Water

IBD: “exposure to fluoride seems indirectly associated with higher incidence of IBD ... Epidemiological studies suggest an association between fluoride exposure and IBD” [157]

ID: “sodium fluoride (NaF) treatment of rats by gavage for 28 days resulted in the induction of oxidative stress and immunotoxicity… NaF treatment lowered cellular immunity in the rats as illustrated by a significant diminution in peripheral blood lymphocyte, monocyte and neutrophil counts in conjunction with a reduction in splenocyte counts. Effects of NaF treatment on humoral immunity were reflected here in a lowering of the levels of plasma IgG specific to a test antigen (i.e., bovine serum albumin). Disorganization in the histoarchitecture was also noted in the host spleen and thymus after NaF treatment.” [158]

##### Mercury

IBD: “In a patient with chronic ulcerative colitis in remission, occupational exposure to mercury vapor led to episodes of disease reactivation.” [159]

ID: “Significantly increased IgE levels was found in the mercury-exposed individuals. … These results suggest that the humoral immune response is an indicator of cellular changes in workers chronically exposed to mercury, even in those with urinary mercury concentrations within levels considered safe in the occupational area.” [160]

COV: “The existing data demonstrate that As, Cd, Hg, and Pb exposure is associated with respiratory dysfunction and respiratory diseases (COPD, bronchitis)…..reduction of toxic metal exposure may be considered as a potential tool for reducing susceptibility and severity of viral diseases affecting the respiratory system, including COVID-19.” [154]

##### Microplastics

IBD: “Numerous animal studies have shown that exposure to nano- and microplastics leads to impairments in oxidative and inflammatory intestinal balance, and disruption of the gut's epithelial permeability. Other notable effects of nano- and microplastic exposure include dysbiosis (changes in the gut microbiota) and immune cell toxicity. Moreover, microplastics contain additives, adsorb contaminants, and may promote the growth of bacterial pathogens on their surfaces: they are potential carriers of intestinal toxicants and pathogens that can potentially lead to further adverse effects. ... Despite the scarcity of reports directly relevant to human, this review brings together a growing body of evidence showing that nano- and microplastic exposure disturbs the gut microbiota and critical intestinal functions. Such effects may promote the development of chronic immune disorders.” [161]

ID: “nano- and microplastic exposure disturbs the gut microbiota and critical intestinal functions. Such effects may promote the development of chronic immune disorders” [161]

##### Nickel

IBD: “demonstrates that UC patients have a significantly higher incidence of hypersensitivity to nickel and palladium, suggesting the possible involvement of dental metal hypersensitivity in UC pathogenesis.” [162]

ID: “Ni toxicity also induces inflammation and several studies demonstrated that Ni could induce immunotoxicity. Excessive Ni exposure can inhibit the development of immune organs by excessively inducing apoptosis and inhibiting proliferation. Furthermore, Ni can decrease T and B lymphocytes, … Ni inhibits the production of cytokines in non-inflammatory responses. Cytokine levels increased in Ni-induced inflammation responses, and Ni activates inflammation through toll like (TL)4-mediated nuclear factor-κB (NF-κB) and signal transduction cascades mitogen-activated protein kinase (MAPK) pathways. Ni has been indicated to inactivate NK cells and macrophages both in vitro and in vivo.” [163]

##### Paraquat

IBD: “catabolism of cysteine by gut microbiota can release high levels of sulfide that may underlie the development or relapse of ulcerative colitis … results suggest that sulfide toxicity is mediated by reactive oxygen species (ROS) … sub-toxic levels of the superoxide generator paraquat can also increase the tolerance of worms to sulfide. Therefore, it appears that activation of ROS detoxification pathway prior to the exposure to sulfide, can increase the tolerance to sulfide toxicity.” [164]

ID: “treatment with 20 mg/kg PQ increased apoptosis of late stage effector cells to yield less memory cells and thereafter impair memory immune response,” [165]

##### PCBs (Polychlorinated Biphenyls)

IBD: “We and other researchers verified that excessively produced free radicals by neutrophils induce various diseases such as Behçet's disease, MCLS, SLE (neutrophil-stimulated lymphocytes), RA (synovial fluid neutrophils), Crohn's disease, colitis ulcerosa, and dermatitis herpetiformis (Dühring). … It was also found that PCB, methyl-Hg and Mn, Cd induce neuropathic diseases through the increase in free radical production.” [166]

ID: “Mixture and concentration-response experiments demonstrated a suppression of phagocytosis by non-coplanar PCBs suggesting a previously unrecognized aryl hydrocarbon receptor (AhR)-independent pathway. … Our results are cause for concern as they suggest an AhR-independent pathway through which non-coplanar PCBs modulate phagocytosis, the immune system's first line of defense, possibly increasing the risk to developing infectious disease.” [167]

##### PFOA (Perfluorooctanoic Acid)

IBD: “we found a strong positive exposure-response relation between PFOA serum levels and subsequent ulcerative colitis (UC) in a high-exposed population from the mid-Ohio valley” [168]

ID: “This study demonstrates that PFOA can affect IL expression level through NF-κB, and ILs have an important function in the mediation of Ig secretion.” [169]

COV:“Measures of individual exposures to immunotoxic PFASs included short-chain PFBA known to accumulate in the lungs. Elevated plasma-PFBA concentrations were associated with an increased risk of a more severe course of COVID-19.” [170]

##### PAH (Polycyclic Aromatic Hydrocarbons)

IBD: “Nicotine, nitrosamines, and polycyclic aromatic hydrocarbons included in tobacco smoke are major causative agents of tobacco smoking-related alimentary diseases. These agents have harmful effects on digestive organ through compromised blood circulation, impaired neural regulation, and cell dysfunction. These malfunctions cause tissue destruction, such as chronic inflammation and ulcer formation. Besides forming DNA adducts, they also act on endogenous signal transduction, leading to gene mutation and abnormal cell death and growth. Interestingly, in inflammatory diseases, smoking improves symptoms of ulcerative colitis, while it worsens the condition of Crohn diseases” [171]

ID: “Ten polycyclic aromatic hydrocarbons were evaluated following 14 days of subchronic exposure in female B6C3F1 mice. … The immunosuppression observed following both subchronic and acute exposure was similar to the structure-activity relationship observed for the carcinogenicity of the compounds tested. … Benz(a)anthracene, benzo(a)pyrene, dibenz(a,c)anthracene, and dibenz(a,h)anthracene suppressed the antibody-forming cell response by 55–91%. The greatest suppression was observed with the 3-methylcholanthrene and 7,12,-dimethylbenz(a)anthracene.” [172]

COV: “polycyclic aromatic hydrocarbons (PAHs) are among the outdoor air pollutants that are major factors in diseases, causing especially adverse respiratory effects in humans. … Evidence supports a clear association between air concentrations of some pollutants and human respiratory viruses interacting to adversely affect the respiratory system. … the association between air pollutants and the transmission and severity of the effects caused by the coronavirus named SARS-CoV-2, which causes the COVID-19. Although to date, and by obvious reasons, the number of studies on this issue are still scarce, most results indicate that chronic exposure to air pollutants delays/complicates recovery of patients of COVID-19 and leads to more severe and lethal forms of this disease.” [173]

##### Silica Dust

IBD: “Silica dust exposure correlates with an increased risk of developing UC, especially in men, and the risk seems to increase with the duration and degree of exposure. Conversely, silica dust exposure correlates positively with the risk of developing CD in women.” [174]

ID: “Silica dust exposure induces autophagy in alveolar macrophages through switching Beclin1 affinity from Bcl-2 to PIK3C3” [175]

COV: “Mineworkers continue to experience high levels of silica exposure. The prevalences of silicosis, HIV and pulmonary TB, remain high. Interstitial lung disease, pulmonary TB, and HIV have all been associated with poorer outcomes of SARS-CoV-2 infections.” [176]

##### Sodium Dichromate Dihydrate

IBD: “In summary, administration of sodium dichromate dihydrate in the drinking water to F344/N rats and B6C3F1 mice resulted in focal ulceration, hyperplasia, and metaplasia in the glandular stomach at the limiting ridge in rats in the 1,000 mg/L group and evidence of increased histiocytic infiltration in the liver (female), duodenum of the small intestine, and/or pancreatic lymph nodes at concentrations as low as 62.5 mg/L” [177]

ID: “respiratory defence and immunologic functions were stimulated or inhibited depending on dose and time of chromium (VI) inhalation” [178]

COV: “Urinary concentrations of chromium, manganese, copper, selenium, cadmium, mercury and lead after creatinine adjustment were found to be higher in severe patients than the non-severe cases with COVID-19. … These results suggest abnormities in urinary levels of the trace metals were tightly associated with the severe illness and fatal outcome of COVID-19.” [179]

##### Titanium Dioxide

IBD: “Our data provide support that TiO2 NP ingestion alters the GI microbiota and host defenses promoting metabolic disruption and subsequently weight gain in mice.” [180]

ID: “Our data suggest that TiO2 nanostructural materials suppress splenocytes proliferation by suppressing Th1 cytokines.” [181]

##### Triclocarban

IBD: “exposure to low-dose TCC exaggerated the severity of colitis. … via gut microbiota-dependent mechanisms. Exposure to TCC increased dextran sodium sulfate (DSS)- and interleukin 10 (IL-10) knockout-induced colitis” [182]

ID: “A significant induction of concentrations of proinflammatory mediator and nitric oxide (NO), accompanied by an upregulated expression of inducible NO synthase gene, was detected in zebrafish embryos exposed to TCC. The transcription of immune-response-related genes, including tnf-α, il-1β, il-4, il-8, and cxcl-clc, was significantly upregulated on exposure to TCC. Furthermore, we found that the exposure of zebrafish embryos to TCC decreased immune cell recruiting in the head. Expressions of nf-κb, trif, myd88, irak4, and traf6 were altered on exposure to TCC. These results demonstrated that exposure to TCC at environmental concentrations significantly affects the expression of immune-response-related genes in zebrafish embryos following oxidative stress and the release of proinflammatory mediators through Toll-like receptor signaling pathway.” [183]

##### Turpentine

IBD: “The effects of an intrathecal NMDA antagonist (AP5) on the behavioral changes induced by colorectal inflammation with turpentine in rats” [184]

ID: “Modified immunoglobulin G glycosylation pattern during turpentine-induced acute inflammation in rats” [185]

#### CATEGORY 5 – PSYCHOSOCIAL/SOCIOECONOMIC

##### Low Socioeconomic Status

IBD: “A significant association between level of neighborhood deprivation and ADs [Autoimmune Disorders] was found. The crude odds were 1.32 (95% confidence interval 1.27–1.36) for those residing in the high-deprived neighborhoods compared to those living in low-deprivation neighborhoods. In the full model, where individual level characteristics were taken into account, the odds of ADs were 1.18 (1.14–1.22) in the most deprived neighborhoods. Certain ADs- … Crohn's disease (1.21) ... -remained significantly associated with high level of neighborhood deprivation after adjustment for the individual-level variables.” [186]

ID: “For the 12-mo vaccination alone, the relative incidence of events (95 % CI) on days 4–12 following immunization was 1.35 (1.31–1.38). We observed a significant relationship between socioeconomic status [SES] and vaccination at 12 mo, with lower SES being associated with a higher relative incidence of events (p = 0.0075).” [187]

COV: “By means of individual-level survival analysis we demonstrate that being male, having less individual income, lower education, not being married all independently predict a higher risk of death from COVID-19 and from all other causes of death.” [188]

##### Posttraumatic Stress

IBD:“autoimmune disorders, including thyroiditis, inflammatory bowel disease, … Trauma exposure and PTSD may increase risk for autoimmune disorders. Altered immune function, lifestyle factors, or shared etiology may underlie this association.” [189]

ID: “Altered gene expression of the innate immune, neuroendocrine, and nuclear factor-kappa B (NF-κB) systems is associated with posttraumatic stress disorder in military personnel” [190]

##### Psychological Stress

IBD: “Acute psychological stress increases small intestinal permeability in humans. Peripheral CRH reproduces the effect of stress and DSCG blocks the effect of both stress and CRH, suggesting the involvement of mast cells.” [191]

ID: “The authors examined whether psychological stress … The stress from the academic examination significantly increased IL-1 beta, IL-6, and IL-10 and decreased IFN-gamma production. These findings suggest that examination stress may increase Th2 cell-mediated humoral immunity and macrophage activities and may decrease Th1 cell-mediated cellular immunity.” [192]

COV: “we evaluate preclinical and clinical literature suggesting that chronic stress-induced hyperinflammation interacts synergistically with COVID-19-related inflammation, contributing to a potentially fatal cytokine storm syndrome. In particular, we hypothesize that both chronic stress and COVID-19-related hyperinflammation are a product of glucocorticoid insufficiency. We discuss the devastating effects of SARS-CoV-2 on structural and functional aspects of the biological stress response and how these induce exaggerated inflammatory responses, particularly interleukin (IL)-6 hypersecretion. We postulate that chronic stress should be considered a significant risk factor for adverse COVID-19-related health outcomes, given overlapping peripheral and central immune dysregulation in both conditions.” [193]

##### Restraint Stress

IBD: “we tested the hypothesis that cold-restraint stress would adversely effect the severity of 2,4,6-trinitrobenzene sulfonic acid (TNBS)-induced colitis in rats, and examined mechanisms for the response. Results indicated that increasing intermittent prior exposures to stress significantly enhanced TNBS-induced colitis severity. An associated stress-induced decrease in colonic mucin glycoprotein content, reduction in goblet cells, and histochemical mucin suggested reduced mucin was a pathogenetic factor.” [194]

ID: “Echinacea purpurea Protects Against Restraint Stress-Induced Immunosuppression in BALB/c Mice” [195]

##### Sexual Abuse

IBD: “However, subjects with irritable bowel syndrome reported much higher rates of childhood sexual abuse and psychosomatic symptoms.” [196]

ID: “Most studies reported an increase of inflammatory activity associated with the presence of early abuse. IL-6, TNF- α, and C-reactive protein were the most frequently analyzed markers and some studies showed higher levels in individuals that suffered CSA compared with controls,” [197]

## Appendix B

### ALTERED BIOMARKERS THAT LINKED CFs TO DISEASE

Table 1 summarized the fifty validated CFs that were common to IBD and immune system dysfunction/COVID-19. Appendix A presented excerpts from the relevant references supporting the linkages between the CFs addressed and the diseases to which they contribute.

In this Appendix, Table 1 is expanded to include the biomarkers that were altered by the CFs to establish their link to the relevant disease(s). For those examples where the excerpts were extracted from the titles or abstracts, biomarkers shown are those listed in the titles and abstracts of the references listed. For those few examples where excerpts were extracted from the full-text, the relevant biomarkers from the full-text excerpts were also included.

Specific biomarkers (*e.g.*, IL-1, CRP, *etc.*) were included wherever possible. More general biomarkers (*e.g.*, inflammation, oxidative stress, mitochondrial dysfunction, *etc.*) were included, especially if specific biomarkers were lacking or minimal. Symptoms (*e.g.*, bleeding, diarrhea, *etc.*) were included when specific or general biomarkers were absent, or minimal. Diseases (CD, UC, *etc.*) were mentioned if biomarkers or symptoms were unavailable.
